# Progress in Surgery

**Published:** 1896-09-05

**Authors:** 


### Progress in Surgery.

### SURGERY OF THE LIVER AND GALL BLADDER.

**Excision of a Portion of the Liver** for tumour has been performed by Mayo Robson[1]. The patient was a woman, aged fifty-four. The operation was performed for a rapidly growing tumour of the gall bladder, which on exposure appeared to be malignant, and as there was only one nodule of the disease evident in the liver, and that in close proximity to the distal end of the cystic duct, it was thought desirable to remove at the same time the portion of liver affected. The mass after removal weighed, without any of the gall bladder, half a pound. Microscopic examination showed the disease to be carcinomatous. The patient made an uninterrupted recovery, and neither suffered from shock, nor had the temperature or pulse rate above normal. If called upon to operate again, the author says he would unite the visceral and parietal peritoneum by stitches, and having in this way shut off the general peritoneal cavity would remove the tumour by means of Paquelin's knife. In the present case he had encircled the growth and protruding part of the liver with an elastic ligature, brought it out at the abdominal wound, then transfixed it with needles, so as to keep the ligature from slipping, and then cut through the liver tissue, half an inch away from the growth. The pedicle was as thick as his wrist, and a granulating surface was exposed after the separation of the slough. He did not think this method applicable if the liver was hidden away under the ribs, but probably in these cases the liver would be enlarged. Peabody[2] narrates an interesting case of operation on a man for supposed hepatic abscess. No abscess was found, but the left lobe of the liver was studded with small whitish dots like minute commencing abscesses or tubercles. An elliptical piece was removed for examination, and the man ultimately made a complete recovery; but whether there was any relation between the operation and his recovery seems doubtful. Abbe[3] also records a case upon which he operated for supposed abscess of the liver, but he found multiple gummata of the liver varying in size from a bean to a walnut. One or two were at the free edge; some were elevated one-third their thickness above the surface of the liver. They were of grayish, almost malignant, appearance, but two excavated scars showed this to be impossible, and subsequent microscopic examination confirmed the supposition that they were gummata. A wedge-shaped piece of liver about one inch by one inch and a-half was removed, including one of the gummata, and the wound was closed. There was no hæmorrhage. Anti-syphilitic treatment was adopted, and the patient recovered. No history of syphilis could be obtained.

**Hepatic Abscess.**—Anderson and Johnson[4] narrate two cases which exemplify different methods of treatment. In one case, where the structures overlying the liver were adherent from extension of the inflammation to the abdominal parietes, a free incision was made; in the other case operation was in two stages across the pleural cavity, viz, resection of rib and suture of the healthy layers of the pleura to each other in the first stage, and then, three days later, incision through the diaphragm into the abscess, with drainage. Both cases recovered. Two other cases are given by Manson and Smith[5] which exemplify the methods. The first was operated on by Manson's method; part of the seventh right rib was excised, and a drainage tube inserted through a large-sized cannula after tapping the abscess. In the second case peritoneal adhesions did not cover the whole surface of the abscess, and the incision opened the peritoneal cavity in the lower part. This necessitated the packing of the wound with a carbolic compress. The right side of the opening into the liver was attached by a suture to the edge of the parietal wound, but the left side was firmly adherent. A tube was inserted, and the opening was carefully packed round. Both cases recovered. Godlee[6] and Lane[7] also refer to similar cases.

**Hydatid Cyst of the Liver.**—Pantaloni[8] reports a rare case of this disease affecting the right lobe of the liver, situated postero-inferiorly. The cyst was incised and partially excised by a median laparatomy, the patient recovering. The author draws the following conclusions: (1) In cases of hydatid cyst of the postero-inferior portion of the right lobe of the liver, non-suppurating, a successful operation may be performed by a laparotomy through the anterior abdominal wall, which permits not only the easy incision and complete emptying of the cyst, but also the resection of much of the cyst wall. (2) If the patient is made to sit up in bed as early as possible after operation, sufficient drainage will be obtained by this means without the necessity of a lumbar incision.

**Cholecystostomy for Lithiasis of the Gall Bladder** has been performed in thirteen cases by Tuffier.[9] In seven of these the gall-bladder was not infected, and all the patients recovered. In the six other cases the gall-bladder was infected, and three of the patients, all women, died—one on the thirteenth day from pneumonia; another, aged sixty-one, from cachexia; and the third from peritonitis. In the last case the gall-bladder had been opened before it was sutured to the abdominal wall. The flow of bile occurred in two cases on the first day; in the others between the first and the third day. In most cases it materially diminished by the eighth day; in only three it lasted more than a month. Temporary cholecystostomy with drainage appears, therefore, to be an excellent method of treatment of this affection. The author considers it more benign than cholecystectomy, or so-called "ideal" cholecystotomy. Wardle[10] removed a stone from the gall bladder of a woman aged sixty-nine, which was two and a-half inches in length. The patient died suddenly twenty-two hours after operation.

**Choledochotomy** for calculi in the common bile duct is the subject of an exhaustive paper by Fenger,[11] who records six cases. He showed by an experiment on one case which died without operation that a small gall stone in a dilated common duct, may by a "ball-valve" action stop the flow of bile into the duodenum. Of the five cases which were operated upon, four recovered. Fluger thinks: (1) In the majority of cases of stone in the common bile duct the duct is dilated and the stone floats in it. (2) Choledochotomy is the operation of choice, and should be employed whenever applicable. (3) Choledochotomy is applicable in the majority of cases, and those cases demanding other operative procedures are rare. Only in a few cases can a stone in the common duct be removed through the gall bladder. He thinks that a metal probe, such as an uterine probe, should be used for sounding the common and hepatic ducts. The group of symptoms upon which the author relies in diagnosing the presence of stone in the common duct are: (1) The gall bladder is atrophied, or, at least, there is an absence of tumour, abscess, or local tenderness in this locality. (2) Intermittent or remittent attacks of jaundice. (Continuous or remittent icterus, persisting for years may be caused by stone or by non-calculous occlusion. If the icterus exists for two or more years without cachexia, it is probably due to stone, because a malignant tumour in the duct or duodenum would cause death in a shorter time.) (3) Colic is present in about 63·7 per cent. of cases. (In tumours it is found absent in about 87·8 per cent.)

(4) The pain is generally localised outside the gall bladder. (5) The pain is frequently of a remittent character. (6) Vomiting is common and sometimes gives relief. (7) The pain is often relieved and the attack brought to an end by changes of position, which probably displace the floating stone. (8) There is found, in most cases, a rapid and considerable loss of weight which cannot be explained by fever, as this is either absent, or only of slight degree. (9) Intermittent or remittent biliary fever ordinarily follows or precedes the attacks of colic and icterus. (It is not yet decided whether this rise in temperature is due to absorption of retained aseptic bile, or whether the microbic invasion from the intestine, which infests the bile, causes inflammation of the wall of the duct, thus making the fever septic. In one of Fluger's cases, in which the attacks of retention were accompanied by marked fever, the bile from the common duct was sterile). Magill and Jourdan[12] also give a useful and interesting epitome of 72 cases of simple and complicated choledochotomies. Of this number 50 recovered, and 22 died, a mortality of 30·55 per cent. If those cases be deducted which were operated upon *in extremis*, the mortality would be reduced to about 20 per cent.

**Gall Stones Causing Intestinal Obstruction.**—Lobstein[13] has collected 92 of these cases, in 61 of which no operation was performed, 50 per cent. of the patients recovering; while of 31 patients operated upon only 12 recovered—a result doubtless depending upon the fact that only the worst cases were submitted to operation, many dying from shock owing to the lateness of the interference. The diagnosis is best made by the early appearance of fæcal vomiting, and the absence of peritonitis or signs of strangulation. Two-thirds of the patients are women, ranging from forty to sixty years of age. The impacted stone is best removed by a longitudinal incision on the free border of the gut.

[1] Lancet, March 14, 1896; and Brit. Med. Journ., March 14, 1896. [2] Med. Rec., New York, Feb. 8, 1896. [3] Ibidem, Feb. 8, 1896. [4] Lancet, Jan. 4, 1896. [5] Ibidem. [6] Brit. Med. Journ., April 18, 1896; and Lancet, April 18, 1896. [7] Ibidem. [8] Amer. Journ. Med. Sci., March, 1896. [9] Med. Week, Mar. 27, 1896. [10] Brit. Med. Journ., Feb. 15, 1896. [11] Amer. Journ. Med. Sci., Feb., 1896. and March, 1896; and Brit. Med. Journ., April 4, 1896. [12] Internat. Med. Mag., March, 1896, and April, 1896.

